# The Relationship Between Soluble Urokinase-Type Plasminogen Activator Receptor (suPAR) Levels and Treatment Response in Patients With Glomerulonephritis: A Single-Center Experience

**DOI:** 10.7759/cureus.47473

**Published:** 2023-10-22

**Authors:** Kubilay İşsever, Hamad Dheir

**Affiliations:** 1 Internal Medicine, Giresun University Faculty of Medicine, Giresun, TUR; 2 Nephrology, Sakarya University Faculty of Medicine, Sakarya, TUR

**Keywords:** disease severity, treatment response, renal failure, supar, glomerulonephritis

## Abstract

Aim

Soluble urokinase-type plasminogen activator receptor (suPAR) is an important protein that is reported to increase in a broad range of inflammatory processes. We aimed to determine whether suPAR is a significant biomarker in glomerulonephritis for distinguishing patients with treatment response from patients without treatment response in our study.

Materials and methods

This was a prospective study in which 117 patients with biopsy-proven glomerulonephritis and 54 healthy individuals without a known chronic disease (control group) were enrolled. A total of 117 patients were divided into two groups: “treatment responsive” and “treatment nonresponsive.” Blood samples were collected from the patients upon their outpatient clinic visits, and the demographical and lab parameters were compared between the groups.

Results

For the patient group consisting of 117 individuals, 56.4% were male, the mean age was 49.6 years, and the mean follow-up duration was 32.2 months. The most commonly diagnosed glomerular disease was focal segmental glomerulosclerosis (FSGS), followed by IgA nephropathy and membranoproliferative glomerulonephritis, respectively. While suPAR levels were significantly higher in the patient group (166.06 ± 127.66 vs. 119.67 ± 70.53 pg/ml, p = 0.001) (suPAR level ± standard deviation), no significant relationship was found between suPAR levels, treatment response status, and disease severity. Besides, there was no significant relationship between suPAR levels and proteinuria levels, BMI of the patients, and the type of immunosuppressive agent used in the treatment and BMI.

Conclusion

Our study showed that suPAR levels could distinguish a patient with glomerulonephritis from a healthy individual, whereas it has no value in predicting the disease progression and treatment responsiveness.

## Introduction

Acute glomerulonephritis (AGN) is a clinical syndrome of acute inflammation of the glomerulus. Typical presenting symptoms consist of a triad of macroscopic hematuria, hypertension, and edema [1]. According to the 2017 joint report of the Turkish Ministry of Health and the Turkish Society of Nephrology (TND), AGNs are the cause of end-stage renal disease (ESRD) in 6.0% of hemodialysis patients and 11.7% of kidney transplant patients [2]. In the USA, approximately 17% of the population currently has renal dysfunction or proteinuria, and glomerular disease is one of the most frequently seen etiologies. Glomerular damage can manifest in a spectrum ranging from asymptomatic microhematuria and albuminuria to rapidly progressive oliguric renal failure [3]. In Europe and Asia, the most common primary glomerular disease is IgA nephropathy, and the most common cause of nephrotic syndrome is membranous glomerulonephritis [2,3]. Regarding the treatment, follow-up might be enough for asymptomatic patients, while angiotensin-converting enzyme inhibitors (ACEI) or angiotensin receptor blockers (ARB) are the first choice in patients with proteinuria >500-1000 mg/day, and immunosuppressive (IS) agents (azathioprine, steroid, cyclophosphamide, cyclosporine, rituximab, etc.) or plasmapheresis may be preferred in symptomatic, progressive cases [4].

Soluble urokinase-type plasminogen activator receptor (suPAR) is the soluble form of the urokinase plasminogen-type activator receptor molecule and has a molecular weight of 20-50 kD. suPAR level parallels the activation level of the immune system and can be found in plasma, urine, blood, serum, and cerebrospinal fluid. suPAR is reported to be a biomarker that indicates disease severity, extent, and aggressiveness [5,6]. Although suPAR was initially thought to be elevated only in focal segmental glomerulosclerosis (FSGS), studies have shown elevated levels in conditions such as cancer, cardiovascular disease, type 2 diabetes, and HIV [7]. suPAR levels have been found to be correlated with pro-inflammatory biomarkers such as tumor necrosis alpha, leukocyte count, and C-reactive protein (CRP) [8]. In studies, suPAR levels were found to be higher in patients with FSGS compared to patients with other types of glomerulonephritis. In addition, patients with relapsed FSGS have been shown to have higher suPAR levels before transplantation and during the post-transplant relapse process [9,10]. It has been reported that suPAR, which has also been investigated as a marker of response to treatment, was found to be higher in steroid-responsive patients with FSGS than in steroid-resistant patients. In this study, the authors claimed that assessing the pre-treatment suPAR levels in patients with primary FSGS may enable us to predict the response rate to steroid treatment [11]. In our study, we aimed to investigate whether suPAR levels are a significant marker between treatment-responsive and non-responsive patients with glomerulonephritis.

## Materials and methods

This study was conducted in Sakarya University Training and Research Hospital, Nephrology Department Outpatient Clinic between 2018 and 2019. This thesis study was approved by the Sakarya University Medical Faculty Ethics Committee (decision date: September 12, 2018, decision No.: 03). This study was supported by Sakarya University Coordinatorship of Scientific Research Projects (Scientific Research Project No.: 2018-2-9-349).

We followed a standard procedure for each patient included in the study. First, an extra tube of blood was collected from patients with biopsy-proven glomerulonephritis who were regularly followed up at our clinic and who agreed to participate in the study by approving the informed consent form. These blood were centrifuged on the same day in the laboratory according to the instructions, and the plasma was numbered into Eppendorf and stored in an Eppendorf container in a -80°C cabinet. When the analysis was about to be performed for collected samples, blood collected for suPAR was removed from the minus -80°C cabinet and kept at room temperature according to the instructions in the kit package insert. After thawing and reaching the ideal temperature, the blood was numbered for sequencing and plated on a plate. suPAR was assayed on a fully automated micro-ELISA (enzyme-linked immunosorbent assay) device (Grifols, Triturus, Barcelona, Spain) using the kit (SinoGeneClon Biotech Human suPAR ELISA Kit, Hangzhou, China). The micro-ELISA assay procedure (pipetting, incubation, washing, and reading) was performed according to the manufacturer's instructions, and the serum levels of suPAR were quantitatively measured by deriving the cut-off and calibration curves. The results were measured and reported in pg/mL. The detection range of suPAR was based on a range of 12-360 pg/mL in accordance with the package insert. Analytical sensitivity of the kit used 3 pg/mL.

The number of patients included in the patient group reached 119. Information such as biopsy diagnoses, demographic characteristics, comorbidities, height and weight, BMI, medication use, and baseline laboratory data were obtained both from the patients verbally themselves during outpatient visits and from the outpatient files used in their follow-up. The laboratory data obtained at the time of biopsy diagnosis were considered "baseline values," and the laboratory values obtained at the time of extra tube blood collection for suPAR were considered "post-treatment" values. Two patients were excluded from the study, one who entered a dialysis program and one who received kidney transplantation during our follow-up period, and the total number of individuals was 117 in the patient group at the end. A total of 54 hospital staff who had no chronic diseases, no history of drug use, and no symptoms were included in the control group voluntarily. The same blood collection procedure of suPAR, along with other lab parameters, was applied to the control group. Of 117 patients, 54 received IS therapy during the follow-up process. Patients who had to receive IS treatment during the follow-up process were considered the “severe disease group,” whereas the rest of the patient group was considered the “mild/moderate disease group.” Besides, those with "complete remission" (proteinuria below 500 mg/day) or "partial remission" (proteinuria below 3.5 g/day) after treatment were considered “treatment responsive.” The rest of the patient group was considered “treatment-resistant.” Subgroup analyses were performed according to the data of these groups. In the “before vs. after” analyses, treatment response was evaluated six months after the collection date of the baseline values. Data were entered into the NCSS (Number Cruncher Statistical System) 2007 (Kaysville, Utah, USA) program.

NCSS program was used for statistical analysis. In addition to descriptive statistical methods (mean, standard deviation, median, frequency, ratio, minimum, maximum), the distribution of the data was evaluated with the Shapiro-Wilk test. For periodic comparisons of quantitative data, the Wilcoxon test was used for non-normally distributed data. The Kruskal-Wallis test was used for comparisons of three or more independent groups of quantitative data, and the Mann-Whitney U test was used for comparisons of two groups. The McNemar test was used for periodic comparisons of qualitative data. The chi-square test was used for comparisons of qualitative data. Spearman's correlation was used to determine the relationship between quantitative data. Significance was evaluated at p < 0.01 and p < 0.05 levels.

## Results

A total of 117 patients in the patient group and 54 patients in the control group were evaluated. The patient group consisted of 56.4% male and 43.6% female patients. The mean age of the patients was 49.64 years; the youngest patient was 21 years old, and the oldest patient was 97 years old. The mean BMI in the patient group was 28.13 kg/m², which was classified as "slightly overweight." The comorbid diseases of the patients included diabetes mellitus in 19 patients (16.4%), hypertension in 58 patients (50%), and cardiovascular disease in eight patients (6.9%). The most common diagnosis of glomerulonephritis was FSGS (23.9%), followed by IgA nephropathy (17.9%) and membranoproliferative glomerulonephritis (17.1%). The mean duration of hospitalization was 8.3 days, with a minimum of 0 and a maximum of 50 days. The follow-up period of the patients ranged between one and 232 months, with a mean of 32.20 ± 38.60 months. In 54 patients who received immunosuppressive treatment, steroids (methylprednisolone) were the most commonly used agent (96.2%). The second most commonly used agent was cyclosporine (30.2%), the third was mycophenolate mofetil (24.5%), the fourth was azathioprine (22.6%), and the fifth was cyclophosphamide (17%).

In Table 1, age, gender, and laboratory parameters of the groups were compared, and proteinuria, creatinine, BMI, uric acid, total cholesterol, and parathormone levels were found to be significantly higher in the patient group, while albumin and estimated glomerular filtration rate (eGFR) values were found to be significantly lower.

**Table 1:** Comparison of the parameters between the groups ^b^ Mann-Whitney U test; ** p < 0.01; N/A: non-applicable; PTH: parathormone; eGFR: estimated glomerular filtration rate; S.D.: standard deviation; Min: minimum; Max: maximum; BMI: body mass index.

Parameters		Control group (n = 54)	Patient group (n = 117)	^b^p
Age (n)	Mean ± S.D.	45.83 ± 15.26, 19-75 (44)	49.71 ± 15.47, 21-97 (47)	N/A
	Min-Max (Median)			
Gender	Female - n (%), male - n (%)	24 (44.4), 30 (55.6)	51 (42.9), 68 (57.1)	N/A
BMI (kg/m^2^)	Mean ± S.D., Min-Max (Median)	26.13 ± 4.19, 18.30-37.10 (26.95)	28.13 ± 5.32, 17.70-40 (27.90)	0.027**
Proteinuria (g/day)	Mean ± S.D., Min-Max (Median)	0.12 ± 0.12, 0.01-0.90 (0.09)	3.17 ± 2.63, 0.42-14.14 (2.25)	0.001**
Albumin (g/dl)	Mean ± S.D., Min-Max (Median)	4.33 ± 0.31, 3.20-5.10 (4.30)	3.52 ± 0.81, 1.20-4.80 (3.70)	0.001**
Creatinine (mg/dl)	Mean ± S.D., Min-Max (Median)	0.72 ± 0.16, 0.38-1.14 (0.71)	1.48 ± 1.34, 0.36-11.58 (1.23)	0.001**
eGFR (ml/min/1.73m^2^)	Mean ± S.D., Min-Max (Median)	107.82 ± 15.69, 63.89-138.65 (109.34)	69.91 ± 35.58, 2.94-149 (66.65)	0.001**
Uric acid (mg/dl)	Mean ± S.D., Min-Max (Median)	4.96 ± 1.33, 2.50-8.10 (4.80)	6.53 ± 1.84, 2-11.40 (6.70)	0.001**
Total cholesterol (mg/dl)	Mean ± S.D., Min-Max (Median)	196.41 ± 41.68, 121-285 (190.50)	248.14 ± 91.84, 63-640 (233)	0.001**
PTH (pg/ml)	Mean ± S.D., Min-Max (Median)	63.91 ± 28.95, 24.5-163 (54.40)	101.19 ± 82, 16.8-516.5 (80)	0.001**

In Table 2, there was no statistically significant difference between suPAR levels in terms of immunosuppressive intake status (p > 0.05). Although there was no statistically significant difference between the suPAR levels in terms of the response to treatment (among 54 patients receiving immunosuppressive treatment), it was found close to significance since p = 0.057 (p > 0.05). The lower suPAR level in the control group compared to the patient group was found to be statistically significant (p = 0.001; p < 0.01).

**Table 2:** Comparison of the suPAR levels between the groups ^a^: Mann-Whitney U test; * p < 0.05; ** p < 0.001; S.D.: standard deviation; Min: minimum; Max: maximum; suPAR: soluble urokinase-type plasminogen activator receptor.

Patient status		N	suPAR (Mean ± S.D.)	Min-Max (Median)	p
Immunosuppressive treatment	No	63	182.58 ± 149.52	83.6-540 (120)	0.605
	Yes	54	146.78 ± 93.87	82.8-540 (118.5)	
Treatment response	No	26	175.15 ± 125.27	85.2-540 (127.5)	0.057
	Yes	28	120.45 ± 35.7	82.8-215 (108)	
Group	Control	54	119.67 ± 70.53	68.9-540 (97.45)	0.001**
	Patient	117	166.06 ± 127.66	82.8-540 (119)	

Other important results from the analyses include significantly improved outcomes in proteinuria and albumin with increased uric acid levels, while creatinine and eGFR values remained similar in “before vs. after” analyses of the patient group. suPAR levels were found not to be associated with proteinuria levels, BMI of the patients, and the type of IS agent used in the treatment (Tables 3, 4) (p > 0.05).

**Table 3:** Association of suPAR with proteinuria in the patient group S.D.: standard deviation; suPAR: soluble urokinase-type plasminogen activator receptor.

Proteinuria (g/day)	Patients (n)	suPAR (pg/ml, mean ± S.D.)	p
0.5-2	58	171.94 ± 130.79	0.288
2-3.5	23	177.62 ± 154.23	
>3.5	36	149.18 ± 103.74	

**Table 4:** Association of suPAR with the used IS agents IS: immunosuppressive; MMF: mycophenolate mofetil; S.D.: standard deviation; suPAR: soluble urokinase-type plasminogen activator receptor.

IS agent	Patients (n)	suPAR (pg/ml, mean ± S.D.)	p
MMF	13	163.38 ± 133.11	0.619
Azathioprine	12	148.39 ± 125.61	
Methylprednisolone	51	148.02 ± 94.35	
Cyclophosphamide	9	137.25 ± 36.29	
Cyclosporine	16	123.29 ± 46.36	

## Discussion

Our study is the first study in Türkiye and one of the few studies in the world evaluating the association of suPAR levels in glomerulonephritis with disease severity and response to treatment. Statistical analysis revealed that there was no statistically significant difference in suPAR levels between patients who responded to treatment and those who did not. However, since the value of p = 0.057 was close to 0.05 in this analysis, this result can be considered as close to significance. When the literature is examined, it is generally reported that suPAR levels decrease with response to treatment in studies investigating the role of suPAR in diagnosis, follow-up, and treatment. In a study by Alachkar et al., a decrease in serum suPAR levels was reported to occur as a result of plasmapheresis and rituximab treatment in patients followed up with the diagnosis of recurrent FSGS after transplantation [12]. In another study by Wei et al., it was reported that suPAR levels decreased with 26 weeks of treatment and a decrease in proteinuria in a group of 70 patients with primary FSGS from North America and 94 patients with primary FSGS from Europe. Moreover, this decrease became more pronounced in complete remission [10]. However, there are also publications, albeit in the minority, suggesting that there is no relationship between suPAR levels and response to treatment or that there is an inverse relationship contrary to expectations. In a group of 109 biopsy-diagnosed patients with primary FSGS, suPAR levels were found to be significantly higher in patients who were sensitive to steroid treatment than in patients who were resistant [11]. In another study, when suPAR levels were examined in patients with childhood tuberculosis, no decrease in levels was found after two months of treatment, and no relationship was found with age, nutritional status, pulmonary/extrapulmonary tuberculosis, or radiologic features [13]. In another study by Jirak et al., some markers were monitored in response to ivabradine treatment given to patients with heart failure, and no change was found in suPAR levels compared to pretreatment [14]. In our study, similar to these results, no relationship was found between response to treatment and suPAR levels in patients with glomerulonephritis. However, it should be re-emphasized that since the value of p = 0.057 is very close to 0.05, the results may be different in studies with a larger number of patients.

In another analysis performed in our study, no significant difference was found between suPAR levels in patients receiving IS therapy and those receiving only symptomatic treatment. This suggests that suPAR is not an effective biomarker for predicting the course and severity of the disease. When we searched the studies in the literature examining the relationship between disease severity and suPAR levels, it was mostly reported that suPAR levels increase with increasing disease severity. In a study by Soltysiak et al., suPAR levels in children with lupus nephritis were found to be higher than those in children with FSGS, which was attributed to the multiorgan involvement in lupus disease and the spread of inflammation throughout the body [15]. In a study by Ahmed et al. in which suPAR levels were evaluated in 114 patients with chronic renal failure, a correlation was found between suPAR levels and disease severity [16]. Similar to our study, a previous study evaluated 43 pediatric patients with nephrotic syndrome and found no significant correlation between immunosuppressive use status, clinical course of the disease, and suPAR levels. Another cohort study evaluating the association of kidney biopsy and some plasma biomarkers suggested that suPAR was not significantly associated with kidney disease progression or death [17]. These controversial results should be clarified by randomized controlled trials with large sample sizes.

When the relationship between suPAR and response to treatment in other diseases is evaluated, Genç et al. reported that suPAR levels were not effective in predicting the severity and prognosis of COVID-19 [18]. Another study reported that suPAR levels declined after successful treatment of chronic obstructive pulmonary disease exacerbations compared to pretreatment [19]. In a study by Schulman et al., high suPAR levels were associated with unsuccessful treatment in patients with pulmonary tuberculosis [20]. Concordantly, in a study by Nusta et al., suPAR levels in 30 patients with acute myeloid leukemia were found to be lower in patients with complete remission compared to other patients [21]. All these studies, along with our study suggesting an increase in patients with glomerulonephritis than normal individuals, prove that suPAR levels increase in most of the inflammatory conditions. However, the role of suPAR in predicting disease severity and treatment response is still unclear.

It is now clearly known that obesity leads to proteinuria and even end-stage renal failure through renal arteriolosclerosis and glomerular growth over time by first increasing eGFR and then decreasing it in the chronic process [22]. Based on this knowledge, rare studies in the literature aiming to determine whether there is an association between suPAR and obesity have shown that there is no association, similar to our study [23].

The small number of patients, the lack of some data, such as missing suPAR levels before treatments in the patient group, and not classifying the FSGS patients as "primary" and "secondary" are among the limitations of our research. Nevertheless, our results will contribute to the literature and may constitute a background for future randomized controlled trials.

## Conclusions

Our study showed that suPAR levels can distinguish patients with glomerulonephritis from healthy individuals, but there was no correlation between suPAR levels and disease severity and response to treatment. As a result of our study, the suPAR molecule was not considered a useful biomarker in the follow-up of patients with glomerulonephritis and in evaluating their response to treatment. Nevertheless, more studies are needed to clarify the relationship between suPAR levels and diagnosis, follow-up, and treatment processes.
